# MRI-Based Classification of Neuropsychiatric Systemic Lupus Erythematosus Patients With Self-Supervised Contrastive Learning

**DOI:** 10.3389/fnins.2022.695888

**Published:** 2022-02-16

**Authors:** Francesca Inglese, Minseon Kim, Gerda M. Steup-Beekman, Tom W. J. Huizinga, Mark A. van Buchem, Jeroen de Bresser, Dae-Shik Kim, Itamar Ronen

**Affiliations:** ^1^Department of Radiology, Leiden University Medical Center, Leiden, Netherlands; ^2^School of Electrical Engineering, Korea Advanced Institute of Science and Technology, Daejeon, South Korea; ^3^Department of Rheumatology, Leiden University Medical Center, Leiden, Netherlands

**Keywords:** Systemic Lupus Erythematosus, magnetic resonance imaging, cohort studies, unsupervised machine learning, neuroimaging

## Abstract

**Introduction/Purpose:**

Systemic lupus erythematosus (SLE) is a chronic auto-immune disease with a broad spectrum of clinical presentations, including heterogeneous neuropsychiatric (NP) syndromes. Structural brain abnormalities are commonly found in SLE and NPSLE, but their role in diagnosis is limited, and their usefulness in distinguishing between NPSLE patients and patients in which the NP symptoms are not primarily attributed to SLE (non-NPSLE) is non-existent. Self-supervised contrastive learning algorithms proved to be useful in classification tasks in rare diseases with limited number of datasets. Our aim was to apply self-supervised contrastive learning on T_1_-weighted images acquired from a well-defined cohort of SLE patients, aiming to distinguish between NPSLE and non-NPSLE patients.

**Subjects and Methods:**

We used 3T MRI T_1_-weighted images of 163 patients. The training set comprised 68 non-NPSLE and 34 NPSLE patients. We applied random geometric transformations between iterations to augment our data sets. The ML pipeline consisted of convolutional base encoder and linear projector. To test the classification task, the projector was removed and one linear layer was measured. Validation of the method consisted of 6 repeated random sub-samplings, each using a random selection of a small group of patients of both subtypes.

**Results:**

In the 6 trials, between 79% and 83% of the patients were correctly classified as NPSLE or non-NPSLE. For a qualitative evaluation of spatial distribution of the common features found in both groups, Gradient-weighted Class Activation Maps (Grad-CAM) were examined. Thresholded Grad-CAM maps show areas of common features identified for the NPSLE cohort, while no such communality was found for the non-NPSLE group.

**Discussion/Conclusion:**

The self-supervised contrastive learning model was effective in capturing common brain MRI features from a limited but well-defined cohort of SLE patients with NP symptoms. The interpretation of the Grad-CAM results is not straightforward, but indicates involvement of the lateral and third ventricles, periventricular white matter and basal cisterns. We believe that the common features found in the NPSLE population in this study indicate a combination of tissue loss, local atrophy and to some extent that of periventricular white matter lesions, which are commonly found in NPSLE patients and appear hypointense on T_1_-weighted images.

## Introduction

Systemic lupus erythematosus (SLE) is a female-predominant auto-immune disease with a broad spectrum of clinical presentations and multi-organ involvement. SLE is characterized by the production and deposition of several autoantibodies, of which more than 20 are associated with damage to the nervous system and 11 are brain-specific (Hanly et al., 2019). The involvement of the central nervous system (CNS) in SLE leads to a series of non-specific neuropsychiatric (NP) manifestations in 12–95% of SLE patients (Ainiala et al., 2001). These NP symptoms widely range in terms of severity and prognostic implications (Schwartz et al., 2019). NP events in SLE can be directly associated with the disease (NPSLE) or can be explained by another etiology (non-NPSLE). NP symptoms are associated with an increased mortality and reduced quality of life within the SLE population (Ramage et al., 2011). The diagnosis of NPSLE is also difficult due to the heterogeneous nature of NP syndromes. According to the American College of Rheumatology (ACR), 19 different syndromes are described in relation to NPSLE patients, and stem from involvement of the CNS: aseptic meningitis, cerebrovascular disease, demyelinating syndrome, headache (including migraine and benign intracranial hypertension), movement disorder (chorea), myelopathy, seizure disorders, acute confusional state, anxiety disorder, cognitive dysfunction, mood disorder and psychosis (Hanly, 2014). The large variation in the attribution of the NP symptoms across studies and institutions highlights the difficulty in unequivocally diagnose NPSLE. In clinical practice, it is important to correctly classify NP events, since the therapeutic approach is defined based on this classification. A study performed in our center reported that about 15% of NP events attributed to SLE (NPSLE) during the first patient evaluation were reclassified after reassessment as non-NPSLE (Magro-Checa et al., 2017). This discrepancy highlights the pressing need for biomarkers that will contribute to more reliably distinguish between NPSLE and non-NPSLE early in the diagnostic process.

Another contributor to the heterogeneity of NPSLE is the multitude of pathomechanisms that underlie brain tissue damage. Two different underlying mechanisms are thought to play a role in the pathophysiology of NPSLE. One is the inflammatory mechanism, where the blood-brain barrier (BBB) or the blood-cerebrospinal fluid (BCSF) barrier is compromised due to presence of pro-inflammatory factors (Gelb et al., 2018). Subsequently, auto-antibodies can enter the brain and trigger an inflammatory process that results in focal or diffuse tissue damage. The second proposed mechanism is the thrombotic or ischemic mechanism, where vascular injury and occlusion are present (Magro-Checa et al., 2016). These two pathomechanisms act independently of one another and can be both present in the same patient. Due to the lack of a diagnostic gold standard, the best strategy so far for diagnosing NPSLE remains a multidisciplinary expert consensus after standardized evaluation of complaints and a complete battery of tests, including brain magnetic resonance imaging (MRI) (Magro-Checa et al., 2017). Despite conventional brain MRI being the method of choice for clinical evaluation of SLE patients experiencing NP events, morphological changes and brain lesions observed in these patients do not clearly correlate with the clinical symptoms and disease outcome, underscoring the clinical-radiological paradox encountered with many NPSLE patients, defined by the presence of lesions in the absence of symptoms of NPSLE or vice versa (Magro-Checa et al., 2018).

Currently, MRI features can only contribute in a limited way in the diagnostic process, mostly in the way of exclusion. Several studies have shown that patients with SLE have more white matter hyperintensities (WMH) and more atrophy and infarcts compared to controls (Ainiala et al., 2005; Appenzeller et al., 2006, 2007; Kozora and Filley, 2011; Luyendijk et al., 2011). These findings *per se*, albeit indicative of robust presence of structural abnormalities in NPSLE, are not useful for the diagnostic process, and basic metrics such as global atrophy and lesion count and lesion load do not lead to a specific diagnosis. It is therefore imperative to further explore neuroimaging biomarkers in the hope of finding markers that can help clinicians differentiate between NPSLE and non-NPSLE patients, and further down the line, also help in the stratification of NPSLE patients based on their clinical phenotype.

Deep learning has been shown to be useful in diagnostic tasks related to clinical neuroimaging data both in diseases with overt brain damage such as stroke, as well as in diseases in which brain alterations are not directly detectable via standard radiological observation (Zhang L. et al., 2020). Deep learning models can extract significant features that are relevant to clinical diagnosis and can distinguish between patient populations even when the brain alterations are not visibly overt (Islam and Zhang, 2018). Classification tasks, however, require a large number of data sets, as well as trained clinicians to generate labels to aid the categorization process. Dementia (Basaia et al., 2019; Jo et al., 2019; Oh et al., 2019; Stamate et al., 2020) and psychiatric disorders (Lin et al., 2018; Durstewitz et al., 2019) have been natural targets for using deep neural networks, as imaging data for these diseases are widely available. NPSLE, on the other hand, is a sub-category of SLE, which in itself is categorized as an orphan/rare disease (prevalence of 1–5 in 10,000, source^1^) and thus the amount of data available is limited. This makes a supervised ML approach impractical for studying brain abnormalities in NPSLE in a single-center study.

Recently, self-supervised learning approaches, which train the model on unlabeled data by providing self-generated labels from the data themselves, have become popular in image classification. In non-medical applications, self-supervised learning approaches were applied in the prediction of the rotation angles of objects (Gidaris et al., 2018), colorization of gray-scale images (Zhang et al., 2016) and solving randomly generated Jigsaw puzzles (Noroozi and Favaro, 2016). Instance-level identity preservation with contrastive learning has proven effective in learning rich representations for classification (He et al., 2019; Tian et al., 2019; Chen et al., 2020). In this context, self-supervised learning approaches are more suitable for dealing with limited data sets, such as the one presented in this work. Self-supervised learning in biomedical imaging has been implemented in several instances, among which screening of 2-dimensional chest x-ray images (Zhang J. et al., 2020), in the evaluation of cardiac time-series data (Kiyasseh et al., 2020), in tissue segmentation of brain lesions (Gonçalves et al., 2014), in segmentation of renal dynamic contrast-enhanced MRI (Huang et al., 2019), in robust and accelerated reconstruction of quantitative and B_0_-inhomogeneity-corrected R_2_* maps from multi-gradient recalled echo MRI data (Torop et al., 2020) and in quality enhancement of compressed sensing MRI of the vessel wall (Eun Di Jang et al., 2020).

In this study, we hypothesized that a self-supervised learning approach would be effective for the classification tasks in our limited patient population, in particular in the distinction between two important diagnostically different SLE patient groups: NPSLE and non-NPSLE patients. To test this hypothesis, we applied a self-supervised method to 3D structural MRI data with the aim of distinguishing and classifying such data for NPSLE and non-NPSLE patients. To provide a benchmark for the ML algorithm presented here, we performed two secondary analyses on the same data set: a standard tissue volumetric analysis of the two patient populations, and a classification of the data sets based on one-class support vector machine (SVM).

## Materials and Methods

### Patient Population

Leiden University Medical Center (LUMC) is the national referral center for SLE patients with NP complaints in the Netherlands. SLE Patients are referred to the outpatient clinic if they present with NP manifestations. In this retrospective study we initially included 216 patients with SLE recorded between May 2007 and April 2015. Of these, 28 patients were excluded because of undefined diagnosis, 3 patients were excluded because of motion artifacts in the MRI scan, 20 patients were excluded because of brain infarcts over 1.5 cm and 2 patients were excluded due to the presence of other diseases (one for a brain tumor and one for a large arachnoid cyst). This resulted in a total of 163 patients included in this study. The medical ethics committee of Leiden-The Hague-Delft approved of the study and all included patients signed an informed consent form.

All patients were admitted to the clinic for a full one-day visit and underwent an identical standardized assessment that included a brain MRI scan (Zirkzee et al., 2012) and a combination of multidisciplinary medical assessments and extensive complementary tests, necessary for deciding whether the NP-events are attributed to SLE (Magro-Checa et al., 2016). Attribution of NP symptoms to SLE was established during a multidisciplinary consensus meeting. This diagnostic process is described in detail previously (Zirkzee et al., 2012). NP events were classified according to the 1999 ACR nomenclature for NPSLE (ACR AD HOC Committee on Neuropsychiatric Lupus Nomenclature, 1999).

During an intake interview, information about gender, age, and SLE disease duration was provided by the patients and verified by their medical records. During the evaluation, SLE activity and damage indexes were scored for each patient: the SLE disease activity was defined using the Systemic Lupus Erythematosus Disease Activity Index 2000 (SLEDAI-2K) (Gladman et al., 2002); SLE irreversible damage was determined through the Systemic Lupus International Collaborating Clinics/American College of Rheumatology damage index (SDI) (Gladman et al., 2000).

### Magnetic Resonance Imaging Protocol

All patients were scanned, according to a standardized scanning protocol, on a Philips Achieva 3T MRI scanner (Philips Healthcare, Best, Netherlands) with a body transmit RF coils and an 8-Channel head receive coil array. The sequence used for this project was a 3D T_1_-weighted gradient echo scan (voxel size = 1.17 × 1.17 × 1.2mm^3^; TR/TE = 9.8/4.6 ms).

### Magnetic Resonance Imaging Preprocessing

All the T_1_-weighted images were registered to a standard brain template, the Montreal Neurological Institute standard template (MNI152), using FNIRT (FMRIB’s non-linear image registration tool) (Woolrich et al., 2009; Jenkinson et al., 2012), using affine registration with 12 degrees of freedom.

### Machine Learning Pipeline Architecture

**FIGURE 1:**
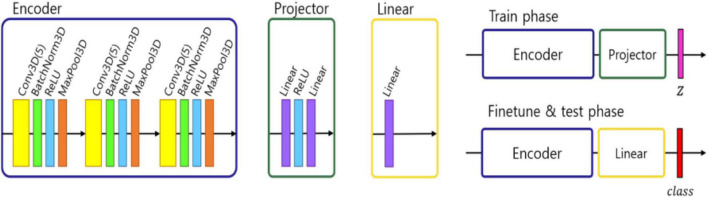
Architecture of ML pipeline: encoder, projector, and linear module. In the training phase, an encoder and projector were used to project the images into representation space with a latent vector z. In the fine tuning and test phase, the projector was changed with a linear module and predict the class.

We followed the self-supervised framework introduced by Chen et al. (2020), where an encoder network *f*_θ_ was used to project the image into a feature space, followed by two-layer multi-perceptron (MLP) *p*_π_ projector that projected the features into latent vector *z*. In our work we modified this approach to address the fact that 3D MRI data required more classification parameters than 2D natural images. Therefore, we designed the encoder network *f*_θ_ using three convolutional layers with batch normalization and a max pooling layer. To test the representation feature, we changed the projector layer into a linear layer which had the same output size as the number of classes, which in our case equals 2 – NPSLE and non-NPSLE. Subsequently, we fine-tuned the linear layer with a training set. Finally, we tested the accuracy of the trained encoder and linear layer. Figure 1 shows the architecture described above.

#### Preprocessing

**FIGURE 2:**
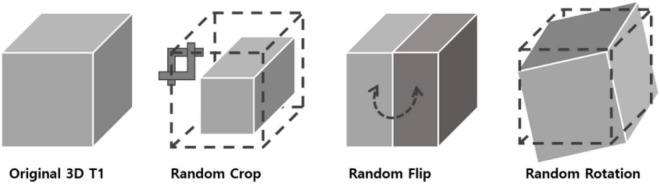
3D data transformations used for data augmentation. Three types of geometrical transformations were applied to the 3D MRI data augment the data set: cropping of the image, left-right flips, and in-plane rotations.

In 2D natural images, Chen et al. (2020) used stochastic data augmentation *t*, randomly selected from the family of augmentations *T*, including random cropping, random color distortion, and random flip. In our 3D MRI data, we used stochastic data augmentation by performing random selection from the set of augmentations we applied to our data. These included: random cropping, random flipping along the z axis and random in-plane rotation. The random crop was applied up to 15 voxels along the three axes. For the random flip, only left-right flips were applied based on the foot-head axis (coronal plane). For rotation, random rotation of angles up to 45 degrees was applied in the left-right, anterior-posterior and foot-head directions. To test the robustness of our method with respect to different data augmentation strategies, we tried three different augmentation settings: one only with crop, one with crop, flip and rotation. The geometric transformations are depicted in Figure 2.

#### Contrastive Loss

The contrastive loss function *L*_*con*_ is defined as follows,


$$L_{con,\theta ,\pi }\left(x,\left\{x_{pos}\right\},\left\{x_{neg}\right\}\right):=-log\frac{\sum_{\left\{z_{pos}\right\}}exp\left(\frac{sim\left(z,\left\{z_{pos}\right\}\right)}{\tau }\right)}{\sum_{\left\{z_{pos}\right\}}\left(\frac{sim\left(z,\left\{z_{pos}\right\}\right)}{\tau }\right)exp+\sum_{\left\{z_{neg}\right\}}exp\left(\frac{sim\left(z,\left\{z_{neg}\right\}\right)}{\tau }\right)}$$

where *z*, {*z*_*pos*_}, and {*z*_*neg*_} are corresponding 128-dimensional representation vectors (*z*) of *x* obtained by the encoder and projector *z* = *p*_π_(*f*_θ_(*x*)). The expression $sim\left(u,v\right)=\frac{u^{T}v}{||u||||v||}$ denotes cosine similarity between two vectors and τ is a temperature parameter (Chen et al., 2020).

We trained the encoder and projector with the contrastive loss function, *NT-xent* which maximizes the similarity between each transformed sample.

#### Patient Selection for Validation of the ML Pipeline

To determine the accuracy of our study, six trials were performed. In each trial, the training set consisted of 68 non-NPSLE and 34 NPSLE patients randomly chosen from within the total patient population (163 subjects). In order to have an equal number of NPSLE and non-NPSLE patients for the training procedure, we used the images of NPSLE twice in every epoch. Therefore, a total of 136 images were used to train the model. In each trial, the test set consisted of 9 non-NPSLE patients and 9 NPSLE patients randomly chosen within the total patient’s population (163 subjects) excluding the training set. The overall demographic and clinical characteristics of the patient population included in this study is shown in Table 1A. The demographic and clinical characteristics of the patients selected in each trial are shown in Tables 1B,C. Detailed age data for the training and test sets in all six trials are given in the Supplementary Tables 1A,B.

**TABLE 1A:** Patient population characteristics for the entire study.

	NP-SLE patients (*n* = 43)	non-NPSLE patients (*n* = 120)
Female, n (%)	37 (86%)	110 (92%)
Age, years	40 ± 13	42 ± 13
NPSLE phenotypes		
*Inflammatory*	30 (70%)	–
*Ischemic*	13 (30%)	–
Hypertension	16 (37%)	35 (30%)
Current smoking	12 (28%)	34 (28%)
BMI	25 ± 6	25 ± 4
Diabetes	3 (7%)	6 (5%)
Duration of SLE, years	6 ± 8	8 ± 8
SLEDAI-2K	8 ± 5	4 ± 4
SDI	1.5 ± 1.2	0.9 ± 1.1

*Sex, age and SLE clinical variables are described for the population included in the study. SLEDAI = Systemic Lupus Erythematosus Disease Activity Index 2000. SDI = Systemic Lupus International Collaborating Clinics/American College of Rheumatology Damage Index.*

**TABLE 1B:** Patient population characteristics for the six training sets.

	Trial 1 (*n* = 102)	Trial 2 (*n* = 102)	Trial 3 (*n* = 102)	Trial 4 (*n* = 102)	Trial 5 (*n* = 102)	Trial 6 (*n* = 102)
Sex (female, %)	90%	91%	91%	93%	90%	89%
Age (mean ± SD)	44 ± 13	41 ± 12	41 ± 12	41 ± 12	41 ± 14	43 ± 13
SLE duration (mean ± SD)	7 ± 8	7 ± 8	7 ± 7	8 ± 8	7 ± 8	7 ± 8
SLEDAI-2K (mean ± SD)	2 ± 2	2 ± 2	2 ± 2	2 ± 2	2 ± 2	2 ± 2
SDI (mean ± SD)	1 ± 1.2	0.8 ± 1	0.8 ± 1	0.8 ± 1	0.9 ± 1.1	0.9 ± 1.1

*Sex, age and SLE clinical variables are described for the population included in each training set of each trial. SLEDAI = Systemic Lupus Erythematosus Disease Activity Index 2000. SDI = Systemic Lupus International Collaborating Clinics/American College of Rheumatology Damage Index.*

**TABLE 1C:** Patient population characteristics for the six test sets.

	Trial 1 (*n* = 18)	Trial 2 (*n* = 18)	Trial 3 (*n* = 18)	Trial 4 (*n* = 18)	Trial 5 (*n* = 18)	Trial 6 (*n* = 18)
Sex (female, %)	100%	89%	94%	89%	100%	100%
Age (mean ± SD)	34 ± 9	46 ± 16	41 ± 10	47 ± 15	38 ± 11	37 ± 13
SLE duration (mean ± SD)	6 ± 7	5 ± 6	9 ± 10	3 ± 5	8 ± 8	7 ± 8
SLEDAI-2K (mean ± SD)	2 ± 1	1 ± 1	2 ± 2	2 ± 2	3 ± 2	3 ± 1
SDI (mean ± SD)	0.83 ± 0.9	1.6 ± 1.5	1 ± 1.2	0.8 ± 1.3	0.7 ± 1.1	0.6 ± 0.8

*Sex, age and SLE clinical variables are described for the population included in each trial. SLEDAI = Systemic Lupus Erythematosus Disease Activity Index 2000. SDI = Systemic Lupus International Collaborating Clinics/American College of Rheumatology Damage Index.*

#### Training Details

**FIGURE 3:**
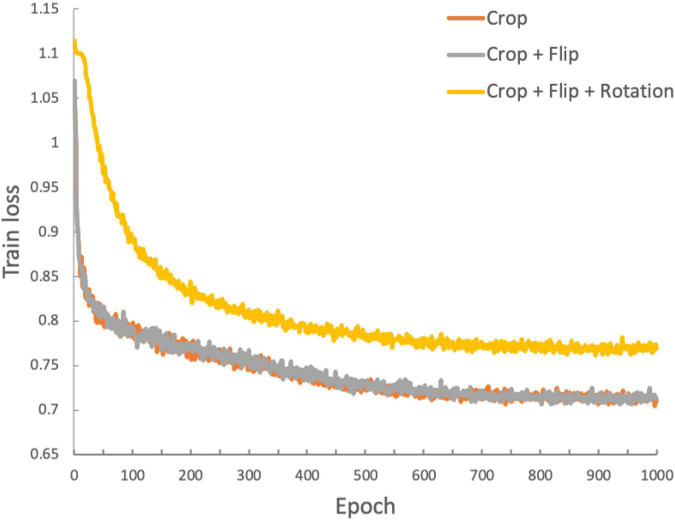
Training loss function for the duration of the training process (1,000 epochs). The three traces represent the loss functions obtained with the three data augmentation strategies: orange: crop only; gray: crop and flip; yellow: crop, flip and rotation.

We used three convolutional layers as the base encoder network and 2-layer multi-layer perceptron with 128 embedding dimensions as the projection head. All models were trained by minimizing the final contrastive loss with a temperature of τ = 0.5. For the rest, we followed similar optimization steps as in SimCLR (Chen et al., 2020). We trained with 1000 epochs under the stochastic gradient descent (SGD) base Layer-wise Adaptive Rate Scaling (LARS) optimizer (You et al., 2017), a cosine annealing learning rate and a gradual warmup scheduler (Chen et al., 2020). We used a weight decay of 1e-6 and momentum of 0.9. We used linear warm-up for the first 10 epochs until a learning rate of 1.0 was achieved and decay with cosine decay schedule. We used a batch size of 16. Furthermore, we used global batch normalization, which shared the parameters over the multiple GPUs. We trained the encoder with contrastive loss. Then, we fine-tuned the encoder along a linear layer to optimize the cross-entropy loss with learning rate 0.001 during 35 epochs. Figure 3 shows the loss curves across the 1,000 training epochs for the three data augmentation strategies. Such intermediate observations are useful for the evaluation of the training process, which in this case appears to be well-behaved for all three strategies.

#### Gradient Class Activation Mapping

There are several methods for interpreting predictions in deep learning (Selvaraju et al., 2016; Doshi-Velez and Kim, 2017; Fong and Vedaldi, 2017; Kim et al., 2017; Shrikumar et al., 2017; Covert et al., 2020). We chose to use gradient class activation mapping (grad-CAM) (Selvaraju et al., 2016). Grad-CAM uses gradient information flowing into the last convolutional layer of the model to assign importance values to each parameter for the prediction of the model. We extended the grad-CAM to deal with 3D convolutional neural networks. Since we are interested in a classification for a specific disease, and images are all aligned to the same space (MNI 152), it was possible to average the grad-CAM maps of test samples to obtain a qualitative measure of the brain regions where significant features were found after averaging across subjects in each trial. For this purpose, for each trial, the grad-cam maps of the samples which were correctly classified in the same class were averaged. Thresholded averaged grad-CAM maps were then overlaid on the MNI template for display.

### Secondary Analyses

We included two secondary analyses to serve as benchmarks to the ML analysis: a volumetric analysis of tissue volumes obtained from segmentation of the T_1_-weighted images, and a one-class supporting vector machine (SVM) analysis.

#### Volumetric Analysis

3D T_1_-weighted images were segmented using the CAT12 toolbox from the statistical parametric mapping software to determine total grey matter (GM), white matter (WM), and cerebral-spinal fluid (CSF) volumes (Inglese et al., 2021). Total intracranial volume was calculated as the sum of gray matter, white matter, and cerebral-spinal fluid volumes. Tissue fractions were obtained by normalizing each tissue volume to the total intracranial volume. Two analyses were performed: (a) a comparison of tissue fractions between the 9 NPSLE and the 9 non-NPSLE subjects included in each trial in the ML analysis, for a total of 6 such comparisons; and (b) comparison of tissue fractions across the entire population of NPSLE (*n* = 43) and non-NPSLE (*n* = 120) included in this study.

### One-Class Support Vector Machine Analysis

For the one-class SVM analysis we used the pipeline provided by the scikit-learn 0.24.2 toolkit^2^. Given the significantly larger size of the non-NPSLE group, we chose it to be class on which the boundaries are defined, and the NPSLE group are tested as belonging to the class or outliers. In a consistent manner with the ML analysis, 68 T_1_-weighted images of non-NPSLE were initially chosen for the classifier to define the class boundaries. Subsequently, randomly chosen 9 NPSLE and 9 non-NPSLE data sets were tested against the classifier. This process was repeated 10 times.

### Statistics

For evaluation of the performance of the algorithm at each trial, we used classification *accuracy, precision and recall* as quantitative measures. An image is considered correctly classified when the model classifies it into the correct class: NPSLE or non-NPSLE. Accuracy was defined as the ratio between the total number of correctly classified samples and the total number of test sets. The coefficient of variation for the three augmentation strategies was also calculated by taking the ratio of the mean accuracy and the standard deviation. Precision was defined as the fraction of correctly predicted NPSLE samples out of the total test samples (NPSLE + non-NPSLE). The recall, defined as the fraction of relevant items selected from the interest class, was calculated as the fraction of correctly predicted NPSLE samples out of the total number of NPSLE samples.

To assess differences in mean accuracy, precision and recall among the three different geometric transformation approaches we used here, repeated-measures analysis of variance (ANOVA) was performed. For the volumetric analysis, the normality of the volume fraction distributions was checked with the Shapiro-Wilk test and value histograms were visually inspected. Differences between NPSLE and non-NPSLE patients across the total population and for the test sets in each trial were assessed with independent sample *t*-tests and *p* values were calculated. All tests were performed using the Statistical Package for the Social Sciences (SPSS) version 25 (IBM corporation, Armonk, NY, United States).

## Results

### Classification Performance

No statistically significant differences were found between NPSLE and non-NPSLE in any of the population characteristics provided in Tables 1B,C (training sets) and (test sets). Table 2A shows the individual and mean accuracy of the classification results for the six trials, defined as the percent of correctly classified NPSLE/non-NPSLE patients out of the total tests within a trial. Results are given for three different strategies for data augmentation that included one (random crop), two (random crop + flip) and three (crop + flip + rotate) transformations. The accuracy (SD) of the six trials in each augmentation strategy was 83.35% (14.05%), 83.33% (16.10%), and 79.63% (11.47%). No significant differences were found when comparing the accuracy of the classification across the three data augmentation options. The coefficient of variation for the three augmentation strategies ranged between 0.14 and 0.19.

**TABLE 2A:** Classification accuracy for the test sets per trial, for all three data augmentation strategies.

Random data augmentation Set	T	Trial 1	Trial 2	Trial 3	Trial 4	Trial 5	Trial 6	Mean (S.D.)
crop	{*T*_1_} ∈ *T*	61.11% (6,5)	77.78% (8,6)	77.78% (5,9)	100.0% (9,9)	88.89% (7,9)	94.44% (9,8)	83.35% (14.05%)
crop, flip	{*T*_1_, *T*_2_} ∈ *T*	61.11% (7,4)	66.67% (6,6)	94.44% (8,9)	83.33% (7,8)	94.44% (8,9)	100.00% (9,9)	83.33% (16.10%)
crop, flip and rotation	{*T*_1_, *T*_2_, *T*_3_} ∈ *T*	66.67% (6,6)	72.22% (8,5)	77.78% (6,8)	100.0% (9,9)	77.78% (8,6)	83.33% (7,8)	79.63% (11.47%)

*Data are shown as percentage. Numbers in parentheses stand for the number of correctly classified non-NPSLE and NPSLE subjects, respectively.*

Table 2B shows the individual and mean precision and recall for the test sets in each of the 6 classification trials. Precision is defined as percent of correctly predicted NPSLE cases out of the total number of tests within a trial, and recall is defined as the fraction of correctly predicted NPSLE samples out of the total number of NPSLE test samples within a trial. The same three sets of geometric transformation for data augmentation were used also here. The precision (SD) across trials was 50.6% (8%), 49.3% (6%), and 48.6% (7%) for trials using only crop, crop + flip, and crop + flip + rotation transformation, respectively. Recall (SD) values were 85.2% (19%), 83.3% (23%), and 79.7% (19%) for trials using only crop, crop + flip, and crop + flip + rotation transformation, respectively. No significant differences were found in accuracy (*p* = 0.531), precision (*p* = 0.845) and recall (*p* = 0.686) among trials using the three different geometric transformations.

**TABLE 2B:** Classification precision and recall for the test sets per trial, for all three data augmentation strategies.

		(precision/recall)						
Random data augmentation Set	T	Trial 1	Trial 2	Trial 3	Trial 4	Trial 5	Trial 6	Mean (S.D.)
crop	{*T*_1_} ∈ *T*	45.5%/55.6%	42.9%/66.7%	64.3%/100%	50.0%/100%	56.3%/100%	44.4%/88.9%	50.6% (8%)/85.2% (19%)
crop, flip	{*T*_1_, *T*_2_} ∈ *T*	36.4%/44.4%	50.0%/66.7%	52.9%/100%	53.3%/88.9%	52.9%/100%	50.0%/100%	49.3% (6%)/83.3% (23%)
crop, flip, and rotation	{*T*_1_, *T*_2_, *T*_3_} ∈ *T*	50.0%/66.7%	38.5%/55.6%	57.1%/88.9%	50.0%/100%	42.9%/66.7%	52.9%/100%	48.6% (7%)/79.7% (19%)

*Data are shown as percentage, and each pair of numbers represents the precision and recall values, respectively.*

An alternative and useful way to assess classifier performance is the receiver operating characteristic (ROC) curve, displaying the true positive rate (recall, or sensitivity) vs. the false positive rate, expressed as complementary to the specificity. We provide the ROC curves for the test sets in all six trials and the three data augmentation strategies within each trial in Supplementary Figure S1 of the Supplementary Material.

### Common Features in NPSLE – Grad-CAM Results

**FIGURE 4:**
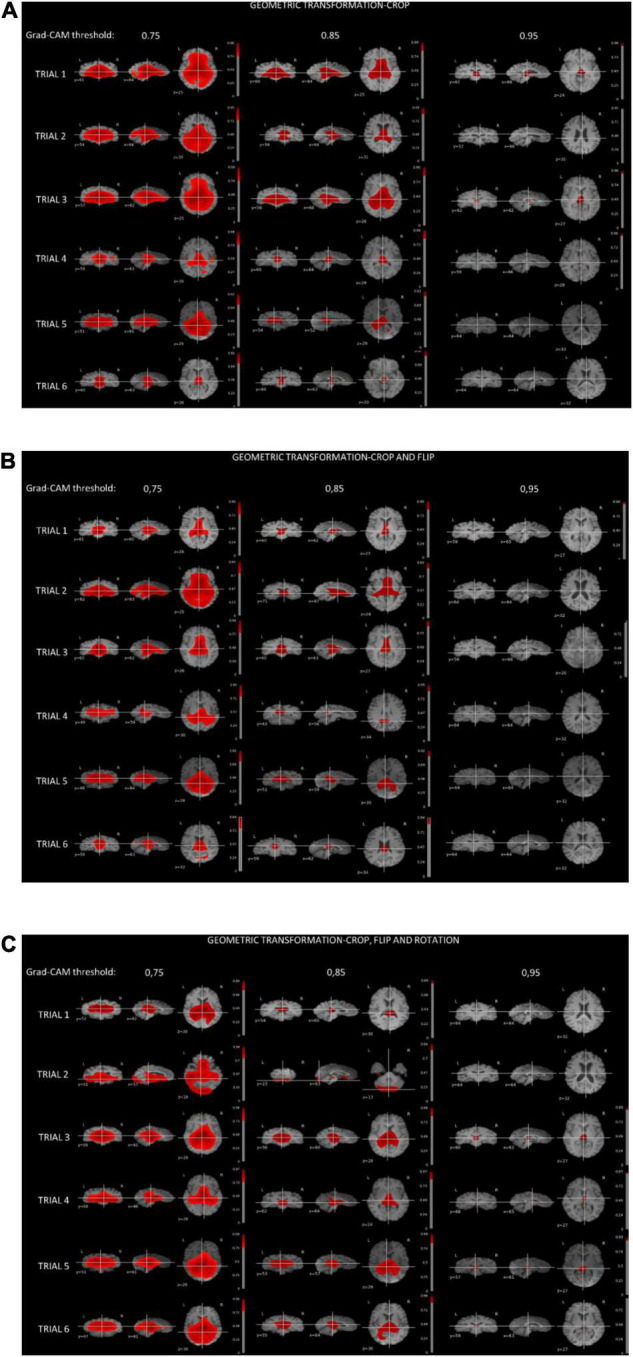
**(A)** Grad-CAM results of NPSLE patients in the six trials augmented with “crop” **(A)**, “crop and flip” **(B)**, and “crop, flip and rotation” **(C)**. Each row displays thresholded mean grad-CAM maps obtained from the NPSLE test set of one of the trials (1 through 6) overlaid on a T1-weighted dataset. Each column displays a different Grad-CAM threshold: 0.75 (left), 0.85 (middle), and 0.95 (right). Within each column, data are shown in three different orientations: coronal (left), sagittal (middle), and axial (right).

**FIGURE 5:**
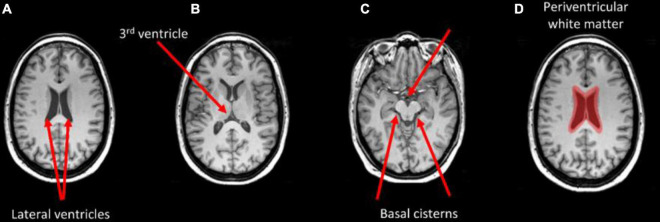
Brain areas that correspond with regions highlighted in the grad-CAM maps with threshold above 0.85. Arrows indicate the lateral ventricles **(A)**, the third ventricle **(B)**, and the basal cisterns **(C)**. Periventricular white matter is highlighted in **(D)**.

Figure 4 shows the thresholded averaged grad-CAM maps for the six trials. Threshold was set at three different levels: 0.75, 0.85, and 0.95. Each of the three panels shows the results following data augmentation with crop only (4A), crop + flip (4B) and crop + flip + rotate (4C). Common features that show up on the grad-CAM maps were found only in the NPSLE cohort. The areas generated by the grad-CAM maps with threshold set at 0.75 are too generic to report on specific brain regions, while the higher thresholds of 0.85 and 0.95 reveal more parcellated maps indicating local involvement. Brain regions showing on the grad-CAM maps with threshold above 0.85 include the lateral ventricles and periventricular white matter, as well as third ventricle and basal cisterns (Figure 5). There were no specific regions that contributed to the model’s prediction of non-NPSLE.

### Secondary Analyses

#### Volumetric Analysis

Table 3 shows the results of the different comparisons of tissue fractions between NPSLE and non-NPSLE: the six comparisons within the test sets and the comparison across the entire population of NPSLE and non-NPSLE subjects. When data are not corrected for multiple comparisons, three comparisons show significant differences between NPSLE and non-NPSLE: GM and CSF in trial 4, and WM in trial 6. These differences become non-statistically significant when the *p* values are corrected for multiple comparisons (6 trials × 3 tissue types = 18 tests) with Bonferroni correction. No statistically significant difference in any tissue fraction was found between the total population of NPSLE patients and that of non-NPSLE patients.

**TABLE 3:** Comparisons of GM, WM, and CSF tissue fractions between NPSLE and non-NPSLE across test sets in all six trials and in the entire study population.

	NPSLE	Non-NPSLE	*p*-value
*Test sets*			
*Trial 1*	*n* = 9	*n* = 9	
GM	0.43 ± 0.02	0.42 ± 0.03	0.139
WM	0.35 ± 0.01	0.35 ± 0.03	0.804
CSF	0.22 ± 0.03	0.24 ± 0.05	0.256
*Trial 2*	*n* = 9	*n* = 9	
GM	0.40 ± 0.03	0.39 ± 0.03	0.441
WM	0.34 ± 0.02	0.34 ± 0.03	0.777
CSF	0.26 ± 0.03	0.27 ± 0.04	0.592
*Trial 3*	*n* = 9	*n* = 9	
GM	0.40 ± 0.02	0.39 ± 0.02	0.417
WM	0.34 ± 0.02	0.34 ± 0.02	0.914
CSF	0.26 ± 0.04	0.27 ± 0.03	0.508
*Trial 4*	*n* = 9	*n* = 9	
GM	0.38 ± 0.04	0.40 ± 0.03	0.366
WM	0.33 ± 0.02	0.35 ± 0.02	0.116
CSF	0.29 ± 0.05	0.26 ± 0.03	0.140
*Trial 5*	*n* = 9	*n* = 9	
GM	0.37 ± 0.03	0.41 ± 0.02	0.023*
WM	0.33 ± 0.01	0.34 ± 0.02	0.050
CSF	0.30 ± 0.04	0.25 ± 0.04	0.019*
*Trial 6*	*n* = 9	*n* = 9	
GM	0.41 ± 0.03	0.40 ± 0.02	0.537
WM	0.33 ± 0.01	0.37 ± 0.03	0.006*
CSF	0.26 ± 0.04	0.24 ± 0.04	0.244
*Total populations*	*n* = 43	*n* = 120	
GM	0.40 ± 0.03	0.40 ± 0.03	0.478
WM	0.34 ± 0.02	0.34 ± 0.03	0.126
CSF	0.26 ± 0.04	0.25 ± 0.05	0.252

Tissue volumes were normalized to total intracranial volume resulting in (unitless) tissue fractions. These are expressed as mean ± SD. Differences between NPSLE and non-NPSLE were calculated with unpaired t-tests and expressed as p values, *p < 0.05. values in bold represent statistically significant differences between the two cohorts.

#### One-Class Support Vector Machine Analysis

Our attempt to apply one-class SVM to distinguish between T_1_-weighted images of NSPLE and non-NPSLE was unsuccessful: all non-NPSLE as well as all NPSLE data sets in all 10 trials were classified as within-class, i.e., belonging to the non-NPSLE group.

## Discussion

In this study, we designed a self-supervised machine-learning pipeline for classification of T_1_-weighted MRI images aimed at distinguishing between images of NPSLE patients and those of non-NPSLE patients. The accuracy of the classification algorithm, based on six repeated trials, was significantly above random choice, and practically independent of the augmentation strategy. The mean classification accuracy into the two classes, NPSLE and non-NPSLE, across the three augmentation strategies ranged between 79 and 83%. Within-augmentation variability across trials was well contained: the coefficient of variation for the three augmentation strategies ranged between 0.14 and 0.19, indicating a good repeatability of the accuracy of the classification, despite the significant heterogeneity of clinical measures, disease phenotypes and the symptoms in the NPSLE population.

The overall precision of our model, representing the fraction of correctly predicted NPSLE out of the total number of NPSLE predictions, was lower, at about 51.8%. This indicates a relatively high rate of false negatives (non-NPSLE subjects identified as NPSLE). This may indicate that the structural brain changes characteristic of NPSLE and were picked up by the classification algorithm can also be found in non-NPSLE patients. MRI abnormalities, such as lesions, local atrophy and other diffuse abnormalities have been found in non-NPSLE and as well as in SLE patients without NP when compared with healthy controls (Ainiala et al., 2005; Appenzeller et al., 2006, 2007; Kozora and Filley, 2011; Luyendijk et al., 2011). It is thus not surprising that some of the features picked up in the training of the algorithm were erroneously attributed to non-NPSLE patients. It remains to be seen whether with increased population size (via, for example, a multicenter effort), or additional MRI modalities that reflect better other aspects of structural changes in the disease (for example, FLAIR T2 images that report on white matter hyperintensities) will contribute to the precision of the classification and limit the false-positives. Conversely, the recall, defined as the fraction of total relevant results (correctly predicted NPSLE patients) out of the NPSLE group, averaged at about 83% indicating a relatively low rate of false negatives, i.e., NPSLE patients that were not classified as such. This corroborates the meaningfulness of the features found by the ML algorithm and provides support to their link to brain changes in NPSLE population.

We studied the relationship between brain alterations in NPSLE and the common features identified by the classification algorithm with grad-CAM, a commonly used visualization tool that provides a coarse localization map highlighting important regions in the image for the classification task. As of recent, Grad-CAM maps have been applied to medical imaging modalities, including a successful application aimed at grading gliomas based on MR images (Pereira et al., 2018; Stoyanov et al., 2018). While grad-CAM maps do not provide quantitative statistical information in the way that statistical parametric maps do, they do indicate communality in the features that led to the classification. In particular, periventricular white matter, lateral ventricles, third ventricle and basal cistern seemed to be features that discriminate NPSLE patients from non-NPSLE. *In vivo* Structural MRI Studies (Muscal and Brey, 2010) and post-mortem histological analyses of brains of NPSLE patients (Brooks et al., 2010) showed significant amount of small focal lesions and white matter hyperintensities concentrating on periventricular white matter, as well as ventricular dilation. Higher occurrence of periventricular and deep WMH lesions was reported also in SLE patients compared to controls but without stratification for NPSLE versus non-NPSLE patients (Hachulla et al., 1998). Overall, despite their common presence in SLE patients with brain involvement, the etiology of periventricular WMH in NSPLE is not well understood, nor is it fully investigated in other diseases with prevalence of periventricular WMH. In older adults, periventricular WMH appear to be associated with impaired cognitive function, in particular with working memory, and are linked to disruption of long distance white matter connections. Some characterization of periventricular WMH in older adults was provided by diffusion tensor imaging and pathological observations and revealed that periventricular WMH are mostly characterized by gliosis and myelin loss (Griffanti et al., 2018). Further investigation on the role of periventricular WMH in NPSLE patients is necessary to confirm their role and their importance in the disease process. Similarly, additional confirmation for the link between the grad-CAM concentration in the ventricular area in the NPSLE population and either ventricular dilation or periventricular WMH needs to be obtained. It is important to note that common features of brain tissue alterations in NPSLE are not only relegated to volume and location but also to the shape of lesions, and in particular that of white matter hyperintensities. The potential relevance of WMH shape in the radiological diagnostic process has been already demonstrated in diabetes (De Bresser et al., 2018), cerebral small vessel disease (Kant et al., 2019) and stroke (Ghaznawi et al., 2021). Evidence for ventricular and periventricular structural differences between NPSLE phenotypes, including between NPSLE and non-NPSLE, have been already given, including in our own studies (Inglese et al., 2021), and it is possible that the features detected by the ML algorithm in the NPSLE population is a combined effect of volume and shape characteristics. Finally, there is no consistent reporting on direct involvement of the basal cisterns *per se* in SLE or NPSLE, barred few case reports (Kawamata et al., 1991; Tsushima and Kubo, 1999). A plausible explanation for the involvement of the basal cisterns is the effect of atrophy, leading to increase in CSF volume in the basal cisterns as well as in the lateral ventricles.

There are several limitations and challenges associated with our study, and we hope to address some of them in our future efforts. Characterization of the overlapping features between the false negatives and “true” NPSLE subjects will certainly require special attention in future studies, with significantly larger populations of SLE and non-NPSLE patients, and with an inclusion of control groups of SLE patients without NP, as well as of healthy controls. One of the most significant limitations of this study is that we could not use the method implemented here to reliably address the link between NP manifestations and structural brain changes in the NPSLE population. This is a question of great importance, and providing means to investigate it will increase the understanding of the pathological mechanisms behind NPSLE as well as aid in diagnosis. The choice of focusing solely on the classification of NPSLE vs. non-NPSLE patients was partly driven by its diagnostic importance, but also made based on the number of patients available for the training and trial sets. Since the ACR1999 criteria apply only to NPSLE, stratification based on NP manifestations or based on NPSLE disease phenotype would have resulted in groups that are too small for a reliable application of the pipeline we developed in this work. For example, stratification of the NPSLE patients to ischemic and inflammatory phenotypes would have resulted in two groups of 30 and 13 patients, respectively, from which training and trial samples have to be in turn chosen. This is an unrealistic scenario for this particularly study. It is hoped that multicenter studies that carefully address harmonization in the diagnostic and in the imaging process will be able to efficiently address this issue.

Despite the fact that there were common regions highlighted by Grad-CAM in the NPSLE group, it is premature to claim at this point that these regions are indeed clinically significant, and a broader investigation is required. In the current investigation we used only one MRI modality, namely T_1_-weighted images, a modality that is highly sensitive to volumetric structural changes, but less sensitive and less specific to lesions, infarcts, microbleeds and hemorrhages, which all result in local hypointensities. Thus, it is imperative to continue the investigation with a more multimodal approach, with additional modalities that will add more sensitivity and specificity to a variety of structural changes commonly found in SLE and NPSLE. From the algorithm perspective, self-supervised machine learning is identity preserving, and it is therefore possible to add a variety of MRI modalities to the process in the hope of significantly improving the classification performance. For example, information on microbleeds is significantly enhanced with the use of T_2_*-weighted images, and white matter hyperintensities are conspicuous on T_2_-FLAIR images.

In deep learning classification tasks, access to a large amount of data sets is essential. The number of samples (overall number of patient data sets) that were included in our study (163) was limited compared to typical numbers of samples used in classification tasks, typically in the tens of thousands of cases (Zhang J. et al., 2020) NPSLE is a rare and highly heterogeneous disease with respect to the variety and severity of symptoms, and most pertinently with respect to the types and spatial distribution of brain abnormalities found in NPSLE and the underlying pathomechanisms responsible for the damage to brain tissue. and it is therefore not a natural target for supervised machine learning based classification. Our results do not categorically exclude the usefulness of supervised deep learning approaches to classification tasks of the kind we performed here, but do provide an impetus for exploring a variety of approaches with the goal of finding the one that suits the most the type and amount data in need of classification. The goal of this study was to establish a retrospective link between (known) diagnosis and structural brain differences between two classes of samples: NPSLE and non-NPSLE patients. Within the limits of a single-center study we benefitted from the maximum number of patients available in the Netherlands, as well as from the most comprehensive diagnostic process for NPSLE (being a national referral center for the disease). When attempting to use a 2 class-wise supervised learning approach, the model diverged, possibly due to the limited number of data sets and the inconspicuousness of the visual features. Based on the fact that self-supervised learning is known to perform well even with a small data sets (Masood et al., 2015; Bai et al., 2019), we opted for self-supervised learning, in the hope that following the classification, common features for two independent classes will emerge, coinciding with the NPSLE and non-NPSLE patient groups. Eventually, the algorithm was only able to find common features within one class of patients (NPSLE), and no common features were found in the non-NPSLE group in our trials. We cannot claim as a certainty that there are no common features to the non-NPSLE group, but the positive result we obtained for the classification of the NPSLE group supports the notion that in our classification task it was more effective to allow the algorithm to learn the visual representation in a self-supervised manner using similarity across images, rather than providing a-priori class labels.

To conclude: we set the stage for classification of brain imaging data of NPSLE and non-NPSLE patients using deep neural networks, achieving relatively high average accuracy across repeated trials. We showed that self-supervised learning is capable of capturing common image features in one class of subjects (NPSLE). This task could not be accomplished with supervised learning, demonstrating that self-supervised machine learning can capture relatively inconspicuous visual information by cross-entropy loss in the MRI images, and may prove advantageous when only a limited number of data sets is available. The method shown here is modular and can accommodate additional imaging modalities to be included in the classification, and can be easily applied to other studies of rare diseases that suffer from similar limitations.

## Data Availability Statement

The data analyzed in this study is subject to the following licenses/restrictions: MRI images that are part of clinical evaluation of patients, and has been anonymized prior to analysis. Requests to access these datasets should be directed to IR, i.ronen@lumc.nl.

## Ethics Statement

The studies involving human participants were reviewed and approved by the Medical Research Ethics Committee of Leiden-The Hague-Delft. The patients/participants provided their written informed consent to participate in this study.

## Author Contributions

FI: image analysis, data curation, and preparation of manuscript first draft. MK: design of machine learning algorithm and application to data, and preparation of manuscript first draft. GS-B: patient clinical data and contribution to final manuscript. MB and TH: contribution to final manuscript. JB: radiological evaluation and preparation of first draft. D-SK: supervision of ML work and contribution to final manuscript. IR: supervision of project as a whole and preparation of first draft. All authors contributed to the article and approved the submitted version.

## Conflict of Interest

The authors declare that the research was conducted in the absence of any commercial or financial relationships that could be construed as a potential conflict of interest.

## Publisher’s Note

All claims expressed in this article are solely those of the authors and do not necessarily represent those of their affiliated organizations, or those of the publisher, the editors and the reviewers. Any product that may be evaluated in this article, or claim that may be made by its manufacturer, is not guaranteed or endorsed by the publisher.

## Footnotes

1
www.orpha.net

2
https://scikit-learn.org/stable/modules/generated/sklearn.svm.OneClassSVM.html
