# Supplementary figures and images for: MRI-Based Classification of Neuropsychiatric Systemic Lupus Erythematosus Patients With Self-Supervised Contrastive Learning
Source: Front Neurosci. 2022 Feb 16;16:695888. doi: 10.3389/fnins.2022.695888 (PMC8889016; doi:10.3389/fnins.2022.695888)

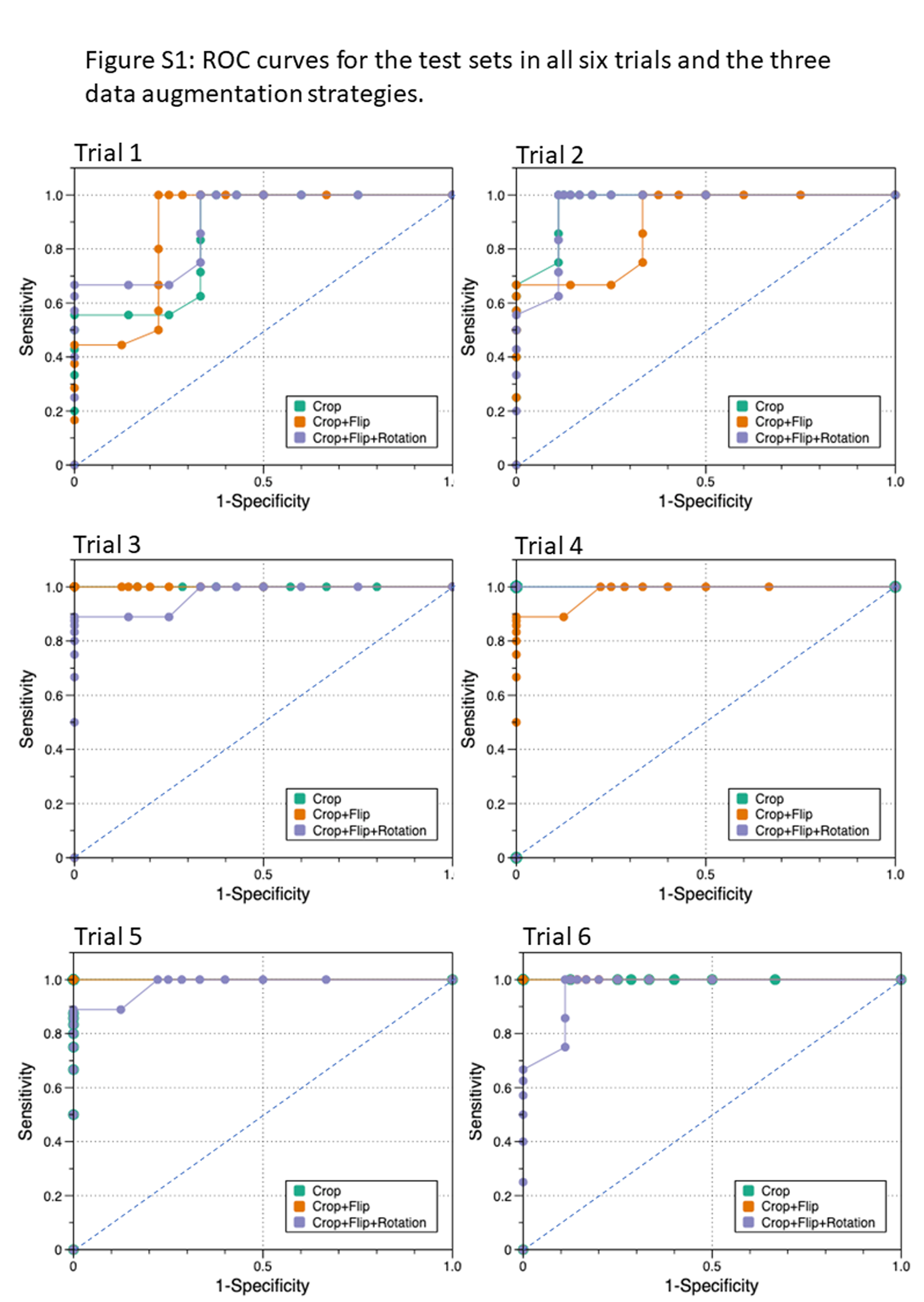

Supplement: Supplementary file 1 [file Image_1.tif]
