# Supplementary material for: MRI-Based Classification of Neuropsychiatric Systemic Lupus Erythematosus Patients With Self-Supervised Contrastive Learning
Source: Front Neurosci. 2022 Feb 16;16:695888. doi: 10.3389/fnins.2022.695888 (PMC8889016; doi:10.3389/fnins.2022.695888)
Supplement: Supplementary file 2 [file Table_1.pdf]

Table S1A Age differences between NPSLE and non-NPSLE for the training set of each trial.

	NPSLE n=34	Non-NPSLE n=68	p-value
**Trial 1**	42±13	45±12	0.238
**Trial 2**	39±13	42±12	0.254
**Trial 3**	40±14	41±11	0.700
**Trial 4**	38±10	42±13	0.111
**Trial 5**	41±14	42±14	0.880
**Trial 6**	42±13	43±13	0.844

Table S1B Age differences between NPSLE and non-NPSLE for the test set of each trial.

	NPSLE n=9	Non-NPSLE n=9	p-value
**Trial 1**	33±10	36±8	0.489
**Trial 2**	44±15	47±17	0.653
**Trial 3**	40±10	43±10	0.481
**Trial 4**	48±19	45±11	0.679
**Trial 5**	37±10	39±13	0.636
**Trial 6**	32±11	41±13	0.112

Age values for each group are expressed as mean ± SD (years). Differences between the two groups are determined with unpaired t-tests.
